# The Epidemiology of Meningitis among Adults in a South African Province with a High HIV Prevalence, 2009-2012

**DOI:** 10.1371/journal.pone.0163036

**Published:** 2016-09-26

**Authors:** Erika Britz, Olga Perovic, Claire von Mollendorf, Anne von Gottberg, Samantha Iyaloo, Vanessa Quan, Verushka Chetty, Charlotte Sriruttan, Nazir A. Ismail, Ananta Nanoo, Alfred Musekiwa, Carl Reddy, Karien Viljoen, Cheryl Cohen, Nelesh P. Govender

**Affiliations:** 1 South African Field Epidemiology Training Programme, National Institute for Communicable Diseases, Johannesburg, South Africa; 2 School of Health Systems and Public Health, Faculty of Health Sciences, University of Pretoria, Pretoria, South Africa; 3 National Institute for Communicable Diseases, Johannesburg, South Africa; 4 Faculty of Health Sciences, University of the Witwatersrand, Johannesburg, South Africa; 5 Division of Global Health Protection, U.S. Centers for Disease Control and Prevention, Pretoria, South Africa; Aga Khan University Hospital Nairobi, KENYA

## Abstract

**Introduction:**

Meningitis is a major cause of mortality in southern Africa. We aimed to describe the aetiologies and frequencies of laboratory-confirmed fungal and bacterial meningitis among adults in a South African province with an 11% HIV prevalence, over 4 years.

**Methods:**

We conducted a retrospective, observational study of secondary laboratory data, extracted on all cerebrospinal fluid (CSF) specimens submitted to public-sector laboratories in Gauteng province from 2009 through 2012. We calculated cause-specific incidence rates in the general and HIV-infected populations and used Poisson regression to determine if trends were significant.

**Results:**

We identified 11,891 (10.7%) incident cases of meningitis from 110,885 CSF specimens. Cryptococcal meningitis, tuberculous meningitis and pneumococcal meningitis accounted for 62.3% (n = 7,406), 24.6% (n = 2,928) and 10.1% (n = 1,197) of cases over the four-year period. The overall incidence (cases per 100,000 persons) of cryptococcal meningitis declined by 23% from 24.4 in 2009 to 18.7 in 2012 (p <0.001) and decreased by 19% among HIV-infected persons from 178.2 to 144.7 (p <0.001). Tuberculous meningitis decreased by 40% from 11.3 in 2009 to 6.8 in 2012 (p <0.001) and decreased by 36% among HIV-infected persons from 54.4 to 34.9 (p <0.001). Pneumococcal meningitis decreased by 41% from 4.2 in 2009 to 2.5 in 2012 (p <0.001) and decreased by 38% among HIV-infected persons from 28.0 to 17.5 (p <0.001). Among cases of other bacterial meningitis (248/11,891, 2.1%), *Neisseria meningitidis* (n = 93), *Escherichia coli* (n = 72) and *Haemophilus influenzae* (n = 20) were the most common organisms identified.

**Conclusions:**

In this high HIV-prevalence province, cryptococcal meningitis was the leading cause of laboratory-confirmed meningitis among adults. Over a 4-year period, there was a significant decrease in incidence of cryptococcal, tuberculous and pneumococcal meningitis. This coincided with expansion of the national antiretroviral treatment programme, enhanced tuberculosis control programme and routine childhood immunisation with pneumococcal conjugate vaccines.

## Introduction

Despite increasing access to antiretroviral treatment (ART) in sub-Saharan Africa, HIV-infected persons have high mortality due to meningitis [1–4]. Common pathogens implicated in meningitis among adults include *Streptococcus pneumoniae*, *Neisseria meningitidis* and in high HIV prevalence settings, *Cryptococcus neoformans* and *Mycobacterium tuberculosis* complex [1]. The high dual burden of HIV infection and tuberculosis in southern Africa has led to a change in the spectrum of causes of meningitis among adults from mostly acute bacterial meningitis to the predominance of cryptococcal meningitis and tuberculous meningitis [1, 5–8]. Cryptococcal meningitis alone causes an estimated 135,300 (95%CI: 91,810 to 188,830) deaths in sub-Saharan Africa annually [9].

Meningitis is potentially preventable and interventions aimed at increasing access to HIV and tuberculosis diagnostics and treatment, as well as strengthening the vaccine programme, have been introduced in South Africa in recent years [10]. An expansion of the public-sector ART programme has resulted in a substantial increase in the number of people receiving ART since 2003, with an estimated ART coverage of 52% by mid-2011 (national ART eligibility threshold of CD4+ T-lymphocyte count <350 cells/μl) [11]. In Gauteng province the number of adults on ART increased from an estimated 188,119 persons in 2009 to 346,351 persons in 2012 [12]. The introduction of improved molecular diagnostic assays for tuberculosis, such as the Xpert MTB/Rif Assay, isoniazid preventive therapy (IPT) and intensified tuberculosis case-detection programmes from 2011, forms part of an enhanced tuberculosis control programme [13, 14]. Implementation of a screening and pre-emptive antifungal treatment intervention for cryptococcal disease started in Gauteng province in September 2012 [15] and was included in the national HIV guidelines in December 2014 [16]. As a strategy to reduce invasive pneumococcal disease (IPD), vaccination of infants with the seven-valent pneumococcal conjugate vaccine (PCV-7) was introduced into the South African Expanded Programme on Immunisation (EPI) in 2009; PCV-13 replaced PCV-7 in 2011 [17].

Understanding the epidemiology of meningitis is necessary to focus public health resources for prevention, early diagnosis and meningitis treatment. Population-level data are also useful to assess the effect of such public health interventions on meningitis. We aimed to describe the aetiologies and frequencies of laboratory-confirmed fungal and bacterial meningitis among adults diagnosed at public-sector facilities, over four years in Gauteng province. We compared trends in incidence and proportions of laboratory-confirmed cryptococcal, tuberculous and pneumococcal meningitis.

## Methods

### Ethics statement

The study protocol was approved by the Faculty of Health Sciences Research Ethics Committee of the University of Pretoria (approval number 428/2014). Permission for the use of secondary data was obtained from the National Health Laboratory Service (NHLS) and data were analysed anonymously.

### Study setting

Gauteng is South Africa’s most densely-populated and economically-active province, with an estimated population of 12.5 million [18] and an HIV prevalence of approximately 11.2% in 2012 [12]. The countrywide coverage for PCV-13 was estimated at 98% in 2012 [19]. The province has 27 NHLS laboratories, the sole service provider for all public healthcare facilities, which serves an estimated 70% to 80% of the provincial population [20, 21]. Healthcare is free of charge or available at a nominal fee in the public sector and 90% of the national population is estimated to have access to a public healthcare clinic within seven kilometres of their home [21].

### Study population and study design

We conducted a retrospective, observational study of secondary laboratory data. Any person ≥18 years who had a lumbar puncture (LP) performed and a cerebrospinal fluid (CSF) specimen sent to an NHLS laboratory, formed part of the population studied. In South Africa, performing an LP is the recommended standard of practice for suspected cases of meningitis [22]. Gauteng province has the highest rate of CSF specimen submission per 100,000 persons compared to other provinces [23].

### Sources of data

Secondary data were extracted from the NHLS Corporate Data Warehouse (CDW). The CDW contains demographic and laboratory data from diagnostic laboratory tests performed by the NHLS. Laboratory personnel enter patient demographic data from test request forms and accompanying laboratory test results into two laboratory information systems (LIS). The electronic records are sent to a central data repository and archived in the CDW. Data were extracted on all CSF specimens submitted to public-sector laboratories in Gauteng from 2009 through 2012. Additional data on tuberculous meningitis, extracted at a different point in time from the same data source, were combined with a master dataset and 88% of these records matched by record-linking (using combinations of patient name, laboratory number and/or date of birth) as no unique identifiers were available. Both linked and non-linked tuberculous meningitis records were included in the analysis. Patient-level HIV test results were not available in this dataset. This is partly because the standard HIV testing algorithm in South Africa recommends that adults are diagnosed using two rapid HIV tests at the point of care (results are thus not captured in the laboratory system unless the rapid test results are discordant and blood is submitted to the laboratory for an HIV EIA) [16]. Additionally, as unique patient identifiers are not used in the South African state health sector, it is very difficult to cross-match data of any (confirmatory) HIV EIAs performed in the laboratory with CSF data.

### Definitions

A case of laboratory-confirmed meningitis was defined as any person ≥18 years with meningitis diagnosed by microbiological testing, as follows. We categorised cases into four groups: 1) Cryptococcal meningitis was diagnosed in a person with a positive India-ink test, a positive cryptococcal antigen (CrAg) test or a positive culture of *Cryptococcus* spp. on CSF. 2) Pneumococcal meningitis was defined as a person with *S*. *pneumoniae* cultured from CSF. 3) A person with *M*. *tuberculosis* complex observed on CSF microscopy (acid-fast bacilli) or CSF culture of *M*. *tuberculosis* or a positive TB-polymerase chain reaction (TB-PCR) (or Xpert MTB/Rif Assay) on CSF was classified as having tuberculous meningitis. 4) Other bacterial meningitis was defined as a person with bacteria other than *S*. *pneumoniae*, which were assessed as potentially pathogenic by the study authors, cultured from CSF. Bacterial latex antigen tests and bacterial PCR assays were not included as diagnostic methods. Mixed infection was diagnosed when a combination of any of the four groups of meningitis was present. Because individuals could have had multiple CSF specimens submitted during the study period, the first positive specimen for each category of meningitis was used to distinguish a new case from duplicate and recurrent cases. Only incident cases of meningitis were included. For cryptococcal meningitis, a duplicate case was defined as a laboratory-confirmed specimen registered on the LIS ≤30 days after the initial laboratory-confirmed diagnosis and a recurrent case any time thereafter. For pneumococcal and other bacterial meningitis, a 21-day interval was used and for tuberculous meningitis the cut-off point for recurrence was 6 months. These definitions were based on laboratory-based surveillance definitions, as previously described [5, 24]. We excluded potential nosocomial and rare pathogens as clinical and other laboratory data (such as CSF cell count/ chemistry or markers of sepsis) were not available to determine if these pathogens caused meningitis. Cases of viral or aseptic meningitis as well as possible bacterial contaminants and records with obvious data entry errors were excluded. Records with missing age and date of birth data were also excluded (~5% of records).

### Statistical analysis

We calculated proportions by dividing the number of cause-specific cases by the total number of laboratory-confirmed cases of incident meningitis. Where appropriate, we used Chi squared and Fisher’s exact tests to compare categorical variables and the Kruskal-Wallis test to compare medians. We calculated the incidence of cryptococcal, pneumococcal and tuberculous meningitis per 100,000 persons by dividing the number of cases of laboratory-confirmed meningitis by annual mid-year population estimates obtained from Statistics South Africa [18]. Incidence of meningitis among HIV-infected individuals was calculated using estimated population denominators from the Actuarial Society of South Africa AIDS and demographic model (ASSA2008), a mathematical model containing age-stratified HIV prevalence estimates at a provincial level, widely used in South Africa for HIV prevalence estimation [12]. We estimated HIV-specific case numbers by multiplying HIV prevalence estimates from published and unpublished laboratory-based surveillance data for cryptococcal and pneumococcal meningitis, as well as tuberculosis estimates from the World Health Organization Global Tuberculosis Report [25, 26] by total case numbers for each type of meningitis. The ASSA2008 model was also used as the source of ART data. We plotted annual cause-specific incidence to assess trends in meningitis over the four-year study period. Poisson regression was used to determine if incidence trends were significant. We calculated the absolute difference between the incidence in 2009 and in 2012, as well as the percentage change in the incidence over four years. STATA, version 13.1 (Statacorp, College Station, TX) was used for data management and all statistical analyses.

## Results

### Spectrum of aetiologies of meningitis among adults

**Fig 1:**
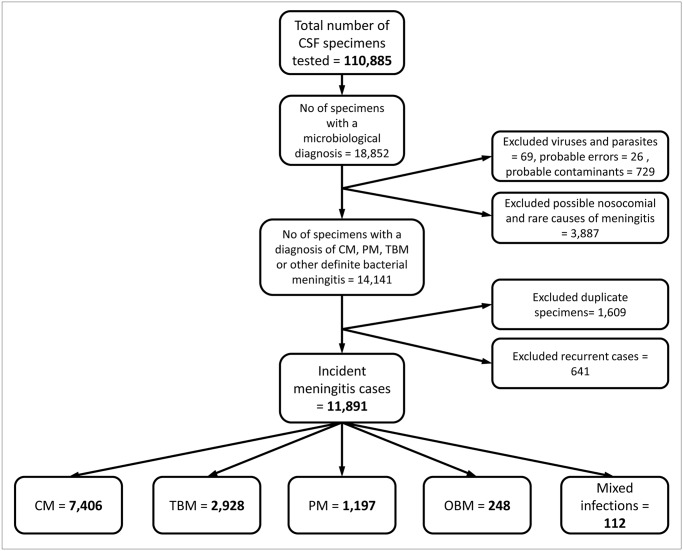
Diagram illustrating cases of meningitis among adults, as extracted from the National Health Laboratory Service Corporate Data Warehouse, Gauteng, South Africa, 2009–2012. CSF = cerebrospinal fluid. CM = cryptococcal meningitis. PM = pneumococcal meningitis. TBM = tuberculous meningitis. OBM = other bacterial meningitis.

During the four-year study period, we identified a total of 11,891 (10.7%) incident cases of laboratory-confirmed bacterial and fungal meningitis from 110,885 CSF specimens tested by NHLS laboratories in Gauteng province. Cryptococcal meningitis accounted for 62.3% (n = 7,406) of all cases of meningitis. Tuberculous meningitis occurred in 2,928 cases (24.6%) and 1,197 cases (10.1%) had pneumococcal meningitis. Other bacterial meningitis comprised 2.1% of the total (248/11,891) (Fig 1).

### Characteristics of study population

**Fig 2:**
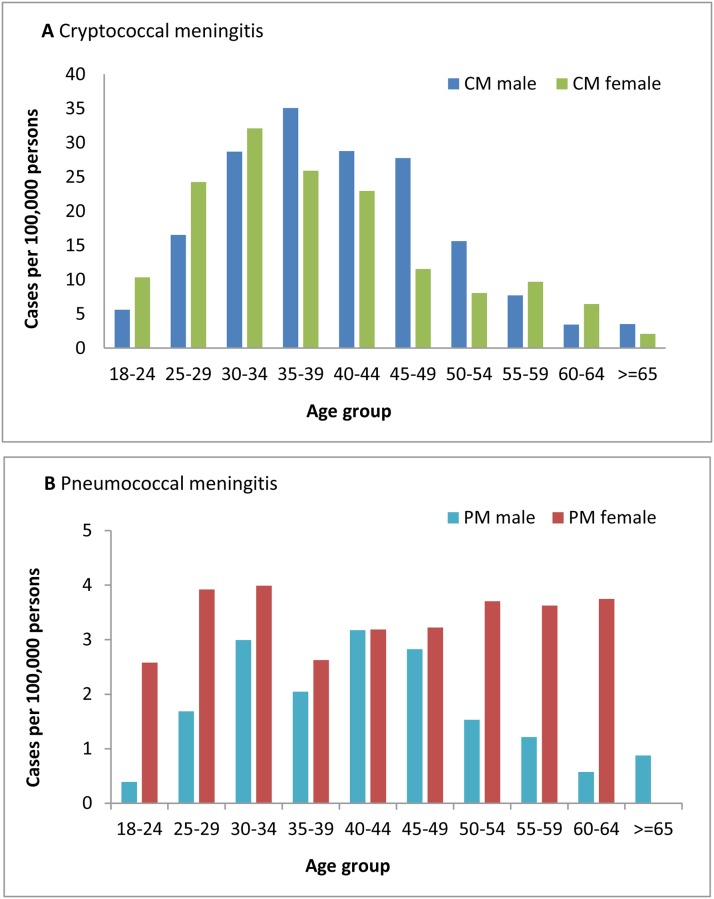
Incidence of cryptococcal meningitis and pneumococcal meningitis, by age group and gender, 2012. CM = cryptococcal meningitis, PM = pneumococcal meningitis.

Among all cases, 11,782 (99.1%) had data on gender and all included cases had either age or date of birth data available. The median age of all cases of laboratory-confirmed meningitis was 37 years (IQR: 30–45) and just over half of all cases were male (50.2%, 5,909/11,782). A statistically significant difference in gender distribution among the four groups of meningitis was observed, with cases of cryptococcal meningitis being predominantly male (52.1%, [3,877/7,444] as compared to 46.8% [2,032/4,338] among cases of tuberculous, pneumococcal and other bacterial meningitis; p <0.001) (Table 1). Among those aged 18–24 years, 25–29 years and 30–34 years, the incidence of cryptococcal meningitis was greater among females than males; however, this ratio was reversed among patients over the age of 35 years. Conversely, cases of pneumococcal meningitis were predominantly female across all age groups, with the exception of the 40–44 year age group, where incidence was similar in males and females (Fig 2).

**Table 1:** Demographic characteristics of patients with laboratory-confirmed fungal and bacterial meningitis in Gauteng province, South Africa, 2009 through 2012 (n = 11,891).

	Cryptococcal meningitis	Pneumococcal meningitis	Tuberculous meningitis	Other bacterial meningitis	Total	p-value
	n (%)	n (%)	n (%)	n (%)		
Age (years)						
Median (IQR)	36 (31–42)	36 (30–45)	36 (30–43)	34 (28–44)	37 (30–45)	0.008^§^
Age (group)						
18–24	471 (6.4)	110 (9.2)	232 (7.9)	41 (16.5)	863 (7.3)	
25–34	2817 (38.0)	412 (34.4)	1058 (36.1)	90 (36.3)	4414 (37.1)	
35–44	2812 (38.0)	381 (31.8)	1068 (36.5)	63 (25.4)	4371 (36.8)	
45–54	971 (13.1)	203 (17.0)	410 (14.0)	37 (14.9)	1636 (13.8)	
55–64	278 (3.8)	73 (6.1)	132 (4.5)	13 (5.2)	499 (4.2)	
≥65	57 (0.8)	18 (1.5)	28 (1.0)	4 (1.6)	108 (0.9)	<0.001°
Gender ^#^						
Male	3815 (52.0)	494 (41.7)	1417 (48.9)	115 (47.5)	5909 (50.2)	<0.001°
District in Province *						
City of Johannesburg	3195 (43.2)	514 (42.9)	1972 (72.7)	137 (55.2)	5896 (50.5)	
City of Tshwane	1327 (17.9)	184 (15.4)	538 (19.8)	50 (20.2)	2124 (18.2)	
Ekurhuleni	1504 (20.3)	280 (23.4)	122 (4.5)	36 (14.5)	1947 (16.7)	
Sedibeng	532 (7.2)	100 (8.4)	27 (1.0)	10 (4.0)	670 (5.7)	
West Rand	847 (11.4)	119 (9.9)	54 (2.0)	15 (6.1)	1038 (8.9)	<0.001°

Totals include mixed infections not displayed.

*451 cases with missing district data.

^#^ 108 cases with missing gender data.

^§^Kruskal-Wallis test.

°Chi squared and Fisher’s exact tests

**Fig 3:**
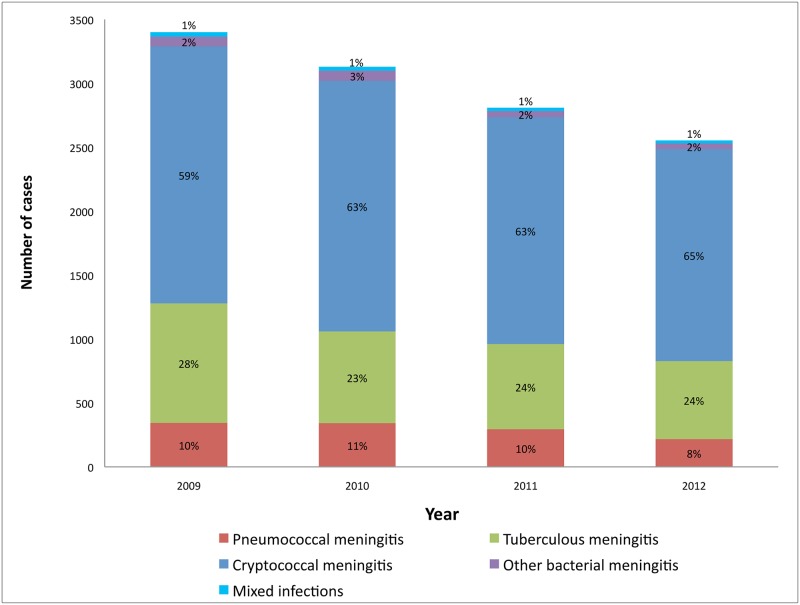
Number of cases and percentages of cryptococcal, tuberculous, pneumococcal and other bacterial meningitis among adults in Gauteng province, South Africa, 2009–2012 (n = 11,891). Mixed infections = a combination of any of the four groups of meningitis.

Table 2 illustrates the major pathogens identified from tested CSF specimens, stratified by year, and shows that the frequencies of all three leading pathogens decreased from 2009 through 2012. Among cases of other bacterial meningitis, the most common organisms identified were *N*. *meningitidis* (n = 93), *Escherichia coli* (n = 72) and *Haemophilus influenzae* (n = 20). There were 17 cases of Group-B *Streptococcus* infection over the four-year period. A total of 112 cases (0.9%) of mixed aetiology occurred, of which 95 cases were cryptococcal and tuberculous, six cases cryptococcal and pneumococcal, five cases of tuberculous and other bacterial (1 *N*. *meningitidis*, 2 *E*. *coli*, 1 *H*. *influenzae*, 1 *Listeria monocytogenes*), five cases of tuberculous and pneumococcal and one case of cryptococcal and other bacterial meningitis (*E*. *coli*). The rate of CSF collection decreased marginally from 2009 through 2011 (320 specimens per 100,000 persons to 313 specimens per 100,000 persons; p = 0.015) and increased in 2012 to 343 specimens per 100,000 persons (p<0.001). When comparing the relative proportions of pneumococcal and tuberculous to cryptococcal meningitis by year, cryptococcal increased relative to the declining proportions of pneumococcal and tuberculous meningitis (Fig 3).

**Table 2:** Number and percentage of major pathogenic organisms isolated from all CSF specimens tested, as recorded in the NHLS CDW, in Gauteng province, South Africa, by year 2009 through 2012 (n = 11,891).

	2009	2010	2011	2012	Total
Organism	n (%)	n (%)	n (%)	n (%)	
*Cryptococcus neoformans*	2010 (59.1)	1961 (62.7)	1776 (63.2)	1659 (65.0)	**7406 (62.3)**
*Mycobacterium tuberculosis*	935 (27.5)	718 (23.0)	666 (23.7)	609 (23.9)	**2928 (24.6)**
*Streptococcus pneumoniae*	344 (10.1)	341 (10.9)	294 (10.5)	218 (8.5)	**1197 (10.1)**
*Neisseria meningitidis*	32 (0.9)	35 (1.1)	18 (0.6)	8 (0.3)	**93 (0.8)**
*Escherichia coli*	18 (0.5)	23 (0.7)	12 (0.4)	19 (0.7)	**72 (0.6)**
*Haemophilus influenzae*	8 (0.2)	4 (0.1)	3 (0.1)	5 (0.2)	**20 (0.2)**
*Listeria monocytogenes*	5 (0.2)	4 (0.1)	3 (0.1)	4 (0.2)	**16 (0.1)**
*Salmonella* non Typhi	5 (0.2)	6 (0.2)	0 (0)	4(0.2)	**15 (0.1)**
Group: B *Streptococcus*	6 (0.2)	4 (0.1)	5 (0.2)	2 (0.1)	**17 (0.1)**
*Streptococcus pyogenes*	3 (0.1)	3 (0.1)	3 (0.1)	0 (0)	**9 (0.1)**
Other streptococci	1 (0.03)	1 (0.03)	3 (0.1)	1 (0.04)	**6 (0.1)**
Mixed infections	33 (1.0)	29 (0.9)	26 (0.9)	24 (0.9)	**112 (0.9)**
**Total**	**3400**	**3129**	**2809**	**2553**	**11891**

### Incidence of meningitis among adults

**Fig 4:**
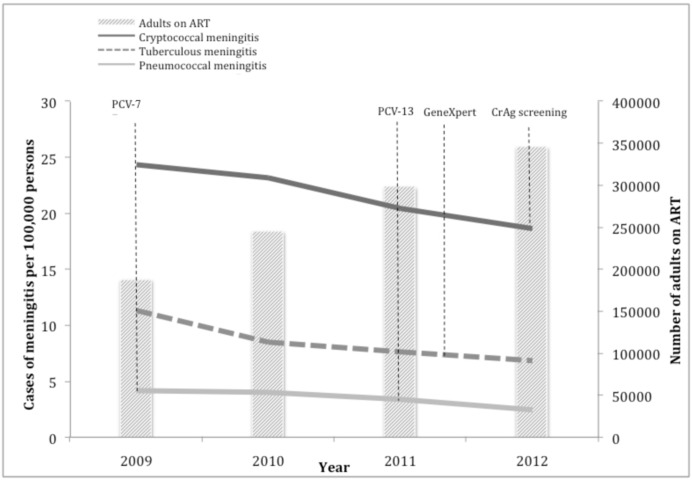
Population incidence of cryptococcal, tuberculous and pneumococcal meningitis among adults in Gauteng province, South Africa, showing key treatment interventions, 2009–2012 (n = 11,531). PCV-7 = seven-valent pneumococcal conjugate vaccine introduction. PCV-13 = thirteen-valent pneumococcal conjugate vaccine introduction. GeneXpert = GeneXpert MTB/Rif assay introduction. CrAg screening = introduction of cryptococcal antigen screening and treatment intervention.

Significant reductions in the incidence of the three major aetiologies of meningitis were observed from 2009 through 2012 (Fig 4). The incidence of cryptococcal meningitis per 100,000 adults decreased by 23% from 24.4 cases in 2009 to 18.7 cases in 2012 (p <0.001). The incidence of tuberculous meningitis decreased by 40% from 11.3 cases in 2009 to 6.8 cases in 2012 (p <0.001). The incidence of pneumococcal meningitis decreased by 41% from 4.2 cases in 2009 to 2.5 cases in 2012 (p <0.001).

The incidence of meningitis decreased among HIV-infected adults in Gauteng province from 2009 through 2012. The incidence of cryptococcal meningitis per 100,000 HIV-infected adults decreased by 19% from 178.2 cases in 2009 to 144.7 cases in 2012. The incidence of tuberculous meningitis decreased by 36% from 54.4 cases in 2009 to 34.9 cases in 2012. The incidence of pneumococcal meningitis declined by 38% from 28.0 cases in 2009 to 17.5 cases in 2012.

## Discussion

In this study in a high HIV-prevalence province of South Africa, cryptococcal meningitis was the leading cause of laboratory-confirmed meningitis among adults, followed by tuberculous and pneumococcal meningitis. The incidence of cryptococcal, tuberculous and pneumococcal meningitis declined significantly among the general and HIV-infected adult populations over the reported period, while the proportion of cases of cryptococcal increased relative to those of pneumococcal and tuberculous meningitis. There was a predominance of males among patients with cryptococcal meningitis and the inverse among cases of pneumococcal meningitis.

These findings are in keeping with previous reports from South Africa [5] and Uganda [27] where cryptococcal meningitis comprised the majority of cases of adult meningitis (63% in Cape Town, South Africa and 60% among HIV-infected individuals in Kampala, Uganda). The number of cases of cryptococcal meningitis was similar to South African national laboratory-based surveillance data collected actively through the GERMS-SA surveillance programme, where a total of 1,973 cases of cryptococcal disease was detected in Gauteng in 2012 [28]. The latter number, however, includes cases below 18 years and cryptococcal disease diagnosed from specimens other than CSF.

The proportion of tuberculous meningitis in 2009 (27.8%) was comparable to the proportion of microbiologically-confirmed tuberculous meningitis cases in Cape Town between 2006 and 2008 (28%) [5]. However, the proportion we found was substantially lower than a similar study in the same setting (GF Jooste Hospital, Cape Town) in 2009 (44%) [29]. The incidence of tuberculous meningitis may have been markedly higher in relation to cryptococcal meningitis in the latter study, as the rate of pulmonary tuberculosis in the hospital’s catchment area exceeded 1500 per 100,000 person-years in 2009. By contrast, the incidence of pulmonary tuberculosis was estimated to be 589 per 100,000 population in Gauteng province in 2009 [30].

The number of cases of pneumococcal meningitis identified by our study (1,197) was smaller than the number of adult cases identified through the GERMS-SA surveillance programme for pneumococcal disease over the four-year period (1,485); however, the latter includes cases diagnosed clinically as meningitis but laboratory-confirmed by blood culture, as well as additional cases confirmed by PCR or Gram stain plus latex agglutination tests.

Although the incidence of all three groups of meningitis declined from 2009 through 2012, the differential decline in pneumococcal and tuberculous meningitis was much larger (41% decline in pneumococcal incidence and 40% decline in tuberculous incidence over 4 years as compared to 23% decline of cryptococcal meningitis). This disparity could be associated with the implementation of multiple interventions for pneumococcal disease during the study period and the commencement of the enhanced tuberculosis control programme in 2011. In contrast, until late 2012, the only intervention possibly contributing to reduction in cryptococcal meningitis was ART. The cryptococcal disease screening and treatment intervention, whereby HIV infected individuals with a CD4+ T-lymphocyte count below 100 cells/μl are reflexively screened for CrAg in blood and if positive, receive pre-emptive antifungal treatment to prevent the development of cryptococcal meningitis, commenced in Gauteng in September 2012 [15]. During this study period, we would likely not yet see the impact of the screen-and-treat intervention. The slower decline of cryptococcal meningitis is not thought to be due to a more sensitive diagnostic test being introduced, as the CrAg lateral flow assay was only introduced to NHLS laboratories after 2012. An increase in the rate of specimen collection in 2012 also highlights that the decreased incidence of meningitis was probably not due to changes in CSF specimen-taking practices.

ART programme expansion likely contributed to the overall decline in meningitis among adults. A change in the ART eligibility criteria for adults from a CD4+ T-lymphocyte count of 200 cells/μl to 350 cells/μl could have contributed to a decrease in extra-pulmonary tuberculosis, as the latter usually occurs in severely immunocompromised persons. This expansion of ART coverage, coupled with earlier detection of tuberculosis through intensified case-finding and improved molecular diagnostics, was suggested as having been instrumental in the decline of microbiologically confirmed pulmonary tuberculosis in Gauteng province and nationwide from 2008 through 2012 [30]. HIV-infected patients have a 33-fold higher risk of developing pneumococcal meningitis than the general population [31]. In our study, a similar decline in incidence of pneumococcal meningitis was found among HIV-infected persons (38%) and the general population (41%). This is in agreement with a South African study that demonstrated similar indirect vaccine effects of PCV vaccination on IPD among HIV-infected and HIV-uninfected adults between 25 and 44 years [17]. A proportion of the decline in incidence of IPD was thought to be due to improvements in ART [17]. The large decrease in pneumococcal meningitis however, could likely be due to PCV vaccination, as substantial reductions in IPD among children and herd effects among adults have been demonstrated [17].

In our study, the overall incidence of cryptococcal meningitis was highest in the 30–39 year age group, mirroring the peak in incidence seen in GERMS-SA surveillance data [28]. The age and gender distribution of cryptococcal meningitis also correlates with the peak population prevalence of HIV-infection in 2012; 30–34 years in females and 35–39 years in males [12, 32]. This is not unexpected, as most cases of cryptococcal meningitis in South Africa (~99%) are associated with HIV infection [25]. Considering a higher burden of HIV infection among women in South Africa, male predominance among persons with cryptococcal meningitis might be even more pronounced. This observation may be explained by health-seeking behaviour of men. Males tend to access healthcare only when in an advanced stage of HIV and rates of ART initiation are lower in men [11]. Therefore male patients are more likely to present with lower CD4 counts and are subsequently at high risk of cryptococcal meningitis. It has previously been demonstrated that South African women with pneumococcal bacteraemia have almost twice as high odds of being HIV-infected than their male counterparts [33]. In this study, 58% of pneumococcal meningitis cases were female. It is not clear if this gender predominance is driven by a biologic propensity or by the disproportionately higher burden of HIV infection among females in South Africa [11, 32]. When stratified by HIV infection status, pneumococcal meningitis appeared to be more common among HIV-uninfected men, whereas among HIV-infected adults, this was more common among women (GERMS-SA, unpublished data). This may suggest that HIV infection drives the female predominance of pneumococcal meningitis in South Africa, although other factors such as severity of immunosuppression may also be important.

Interventions aimed at reducing cryptococcal and tuberculous meningitis should be prioritised, considering their relative contributions to the overall burden of meningitis. Screening for cryptococcal antigenaemia and pre-emptive antifungal treatment among ART-naïve adults has been demonstrated to significantly reduce all-cause mortality in a randomised-controlled trial [34] and has been recommended for implementation across South Africa [10, 16, 27, 35, 36]. Some authors have suggested that diagnostic algorithms for suspected adult-onset meningitis in high HIV-prevalence settings should include point-of-care serum/plasma/capillary finger prick CrAg testing prior to performing a LP, so that CSF opening pressure is measured [27, 35, 36]. The cost-effectiveness of such algorithms has been demonstrated [8]. Mycobacterial culture and microscopy are known to lack sensitivity and diagnosis of tuberculous meningitis is often made on clinical grounds [1, 37, 38]. Although Xpert MTB/Rif offers much faster diagnosis than mycobacterial culture with higher sensitivity than microscopy, its sensitivity may vary from below 50% [39] to above 80% in centrifuged samples of HIV-infected individuals [40]. Xpert MTB/Rif is also an expensive test [40] and as currently implemented, has not been shown to improve care or impact on mortality [13]. This highlights the need for improved low-cost point-of-care diagnostics for tuberculous meningitis and/or better linkage of laboratory results to the clinical setting. Continued emphasis should be placed on further expansion of the nationwide ART programme and ART for all HIV-infected adults [41], with efforts to ensure access to treatment, retention of patients in ART care and early detection of opportunistic infections.

Our study has several limitations. First, the ecologic nature of this study limits causal inferences that can be drawn from the results. However, population-based studies can indicate disease patterns in a community and provide a base from which more in-depth studies can be conducted. Second, as only laboratory-confirmed cases of meningitis from public healthcare facilities were included, we are likely to under-estimate the true disease burden. A proportion of patients with meningitis might have died at home without seeking care and laboratory diagnosis would have been dependent on the taking of CSF specimens at health care facilities. It is possible that we may have over-estimated the incidence of tuberculous meningitis by at least 12%, as we did not have sufficient information to determine if non-linked tuberculosis records represented duplicate cases or not. Nonetheless, as tuberculous meningitis especially, is under-diagnosed by laboratory testing methods alone [38], this analysis still likely represents a minimum estimate of the true tuberculous meningitis burden. We were unable to determine if the proportion of CSF samples that had mycobacterial culture requested had changed over time. This may also have influenced trends with respect to the incidence of tuberculous meningitis. The aetiologies of meningitis in the South African private care setting might also be different from the epidemiology seen in the public sector. Additionally, our data extract excluded CSF cell count and chemistry parameters, limiting further interpretation of CSF results and potentially excluding a proportion of culture-negative meningitis cases. Third, the use of secondary data was limited to existing variables available in the LIS and was in turn dependent on data recorded on laboratory request forms by clinicians at source. Incomplete data may lead to selection bias. The extraction of data from the CDW posed unique challenges in a period of change from one LIS to another across NHLS laboratories at different times commencing at the end of 2011. Inconsistencies in laboratory-recorded specimen and test codes could have led to an over- or under-representation of specimens in the extracted dataset. Finally, patient-level data on HIV infection status were not available for cases in this dataset and modelled HIV prevalence estimates were used to estimate incidence among HIV-infected persons. This may, therefore, not be a true reflection of the incidence of meningitis among HIV-infected adults. Our study also has some strengths in that we had large numbers of specimens from a centralized LIS. In addition, there are few large population-based studies examining the aetiologies of meningitis in sub-Saharan Africa. This study provides a province-wide report.

## Conclusions

Prevention of meningitis is vital in high HIV-prevalence settings. Cryptococcal meningitis was the most common cause of laboratory-confirmed meningitis among adults in Gauteng. The decrease in incidence of all three major causes of meningitis coincides with a period of ART programme expansion, enhanced tuberculosis control and routine childhood conjugate pneumococcal vaccination. Consideration should be given to the inclusion of point-of-care CrAg testing in diagnostic algorithms for adult meningitis, investment in research to find better diagnostics for tuberculous meningitis and emphasis on early ART. Comprehensive studies are needed to explore the impact of interventions on meningitis among HIV-infected persons, as well as national trends.

## Supporting Information

S1 Table Possible nosocomial and uncommon* bacterial and fungal causes of meningitis isolated from CSF culture specimens among adults in Gauteng Province, 2009–2012. Possible nosocomial and uncommon organisms do not usually cause community-acquired meningitis but may cause meningitis due to the consequences of neurosurgical procedures or dissemination following bacteraemia. As clinical and other laboratory parameters were not available, we were unable to determine if these organisms were significant in causing meningitis.
(DOCX)
