# Supplementary material for: The Epidemiology of Meningitis among Adults in a South African Province with a High HIV Prevalence, 2009-2012
Source: PLoS One. 2016 Sep 26;11(9):e0163036. doi: 10.1371/journal.pone.0163036 (PMC5036788; doi:10.1371/journal.pone.0163036)
Supplement: S1 Table — Possible nosocomial and uncommon organisms do not usually cause community-acquired meningitis but may cause meningitis due to the consequences of neurosurgical procedures or dissemination following bacteraemia. As clinical and other laboratory parameters were not available, we were unable to determine if these organisms were significant in causing meningitis. (DOCX) [file pone.0163036.s001.docx]

**Supplementary Table: Possible nosocomial and uncommon* bacterial and fungal causes of meningitis isolated from CSF culture specimens among adults in Gauteng Province, 2009-2012**

| Organism | Number of cases |
| --- | --- |
|  |  |
| *Acinetobacter species* | 174 |
| *Aeromonas species* | 2 |
| *Alcaligenes faecalis* | 1 |
| *Candida species* | 5 |
| *Chryseobacterium indologenes* | 2 |
| *Citrobacter species* | 6 |
| *Coagulase negative staphylococci* | 2252 |
| *Empedobacter brevis* | 3 |
| *Enterobacter species* | 41 |
| *Enterococcus species* | 90 |
| *Escherichia vulneris* | 3 |
| *Flavimonas oryzihabitans* | 1 |
| *Klebsiella species* | 84 |
| *Leclercia adecarboxylata* | 1 |
| *Morganella morganii* | 1 |
| *Pantoea species* | 4 |
| *Proteus species* | 12 |
| *Providencia species* | 5 |
| *Pseudomonas species* | 69 |
| *Saccharomyces species* | 2 |
| *Serratia species* | 12 |
| *Sphingobacterium species* | 1 |
| *Sphingomonas paucimobilis* | 4 |
| *Staphylococcus aureus* | 120 |
| *Staphylococcus epidermidis* | 26 |
| Other *Staphylococcus species* | 22 |
| *Stenotrophomonas maltophilia* | 6 |
| Other Yeasts not identified | 911 |
| Gram negative bacilli not identified | 22 |
| *Nocardia species** | 1 |
| *Pasteurella species** | 1 |
| *Rhodotorula rubra** | 3 |
| Total | **3887** |

**Note:** Possible nosocomial and uncommon organisms do not usually cause community-acquired meningitis but may cause meningitis due to the consequences of neurosurgical procedures or dissemination following bacteraemia. As clinical and other laboratory parameters were not available, we were unable to determine if these organisms were significant in causing meningitis.
